# Transauricular embolization of the rabbit coronary artery for experimental myocardial infarction: comparison of a minimally invasive closed-chest model with open-chest surgery

**DOI:** 10.1186/1749-8090-7-16

**Published:** 2012-02-13

**Authors:** Konstantinos Katsanos, Sofoklis Mitsos, Efstratios Koletsis, Vassiliki Bravou, Dimitris Karnabatidis, Fevronia Kolonitsiou, Athanassios Diamantopoulos, Dimitrios Dougenis, Dimitris Siablis

**Affiliations:** 1 Department of Interventional Radiology, Patras University Hospital, School of Medicine, 26504, Rion, Greece; 2 Department of Cardiothoracic Surgery, Onassion Cardiac Surgery Center, Athens, Greece; 3 Department of Cardiothoracic Surgery, Patras University Hospital, School of Medicine, 26504, Rion, Greece; 4 Department of Anatomy, Patras University, School of Medicine, 26504, Rion, Greece; 5 Department of Microbiology, Patras University Hospital, School of Medicine, 26504, Rion, Greece

**Keywords:** endovascular, coil, heart, transcatheter, left anterior descending, animal study, experimental, myocardial ischemia, embolization, ligation

## Abstract

**Introduction:**

To date, most animal studies of myocardial ischemia have used open-chest models with direct surgical coronary artery ligation. We aimed to develop a novel, percutaneous, minimally-invasive, closed-chest model of experimental myocardial infarction (EMI) in the New Zealand White rabbit and compare it with the standard open-chest surgical model in order to minimize local and systemic side-effects of major surgery.

**Methods:**

New Zealand White rabbits were handled in conformity with the "Guide for the Care and Use of Laboratory Animals" and underwent EMI under intravenous anesthesia. Group A underwent EMI with an open-chest method involving surgical tracheostomy, a mini median sternotomy incision and left anterior descending (LAD) coronary artery ligation with a plain suture, whereas Group B underwent EMI with a closed-chest method involving fluoroscopy-guided percutaneous transauricular intra-arterial access, superselective LAD catheterization and distal coronary embolization with a micro-coil. Electrocardiography (ECG), cardiac enzymes and transcatheter left ventricular end-diastolic pressure (LVEDP) measurements were recorded. Surviving animals were euthanized after 4 weeks and the hearts were harvested for Hematoxylin-eosin and Masson-trichrome staining.

**Results:**

In total, 38 subjects underwent EMI with a surgical (n = 17) or endovascular (n = 21) approach. ST-segment elevation (1.90 ± 0.71 mm) occurred sharply after surgical LAD ligation compared to progressive ST elevation (2.01 ± 0.84 mm;p = 0.68) within 15-20 min after LAD micro-coil embolization. Increase of troponin and other cardiac enzymes, abnormal ischemic Q waves and LVEDP changes were recorded in both groups without any significant differences (p > 0.05). Infarct area was similar in both models (0.86 ± 0.35 cm in the surgical group vs. 0.92 ± 0.54 cm in the percutaneous group;p = 0.68).

**Conclusion:**

The proposed model of transauricular coronary coil embolization avoids thoracotomy and major surgery and may be an equally reliable and reproducible platform for the experimental study of myocardial ischemia.

## Introduction

Ischemic coronary artery disease is a major cause of morbidity and mortality. Appropriate animal models are essential in order to investigate the mechanisms of myocardial infarction and develop new therapeutic interventions. During the last years, experimental myocardial ischemia (EMI) has been one of the most extensively studied topics in modern cardiovascular research. A large body of evidence has been amassed regarding the pathophysiology, pharmacological treatment strategies and relevant interventional therapy in the setting of acute and chronic myocardial ischemia and infarction [1-3]. Experimental coronary infarction and ischemia of the myocardium may be produced in many animal species and in various ways. Most animal studies on EMI have employed the traditional open-chest platform with thoracotomy and direct surgical ligation of the left coronary artery. Open-chest procedures allow direct access to the heart with visual inspection of procedural results, while immediate contact to the epicardial coronary vessels provides the opportunity for accurate measurements of coronary blood flow and other relevant hemodynamic parameters [4,5].

However, open-chest EMI is associated with local and systemic side effects of major surgery. The preceding pericardial incision bears little pathophysiological relevance to human clinical afflictions and may disturb the progression of myocardial remodelling, whereas major surgery alone may affect whole body homeostasis and alter local and systemic immunological and inflammatory responses [6,7]. Alternative closed-chest models of EMI that mainly use endovascular catheterization techniques have been developed, where transcatheter access to the coronaries is typically gained via surgical cutdown and exposure of the carotid or femoral artery. Closed-chest models of EMI primarily avoid the major trauma of thoracotomy with its potential influence on cardiac and whole-body physiology and recovery. Most importantly, they may induce more physiologically more clinically relevant myocardial ischemia [3]. The aim of the present study was to establish a closed-chest, solely percutaneous, minimally invasive technique in order to induce EMI by transauricular embolization of the left coronary artery of the rabbit heart. This model of percutaneous transauricular EMI in the rabbit was compared with the gold standard of experimental myocardial infarction as induced by coronary ligation with a plain suture.

## Methods

New Zealand White rabbits weighing 2.5-3.5 kg were kept in separate cages in an environmentally controlled animal research facility. Food and water were provided ad libitum. This investigation was carried out in conformity with the "Guide for the Care and Use of Laboratory Animals" published by the National Institutes of Health (NIH publication No. 86-23, revised 1985) and was approved by the local Hospital's Scientific and Ethics Committee. All EMI procedures were performed with the animals under dissociative anesthesia with a mixture of ketamine (35 mg/kg) and xylazine (5 mg/kg) i.m. [8]. The experimental protocol consisted of two groups. Group A underwent EMI with an open-chest method involving surgical tracheostomy, a mini median sternotomy and left anterior descending coronary artery ligation with a plain suture. Group B underwent EMI with a closed-chest method involving transauricular superselective catheterization of the left anterior descending coronary artery and distal percutaneous embolization with the introduction of a micro-coil.

### Surgical open-chest technique

After induction of dissociative anesthesia as described previously, subsequent anesthesia was maintained with low doses of propofol. The surgical procedure was performed under aseptic conditions. The rabbits were placed in the supine position. A standard 22-gauge intravenous catheter was inserted into the marginal auricular vein to establish intravenous access. Prophylactic antibiotics (cefuroxime 25 mg/kg i.m.) were administered before and after surgery. The skin was clipped and shaved and standard electrocardiography (ECG) electrodes were attached in both the front limbs and bilateral sides of the lower abdomen. Arterial oxygen saturation was measured with a pulse oximeter. ECG signs, systemic blood pressure and pulse oximetry were monitored continuously during EMI induction. A surgical tracheostomy was performed and the rabbit was ventilated with room air through a 3.5-mm pediatric tracheal tube at a rate of 35-40 breaths per minute and a tidal volume of 10-15 ml using room air enriched with oxygen.

**Figure 1:**
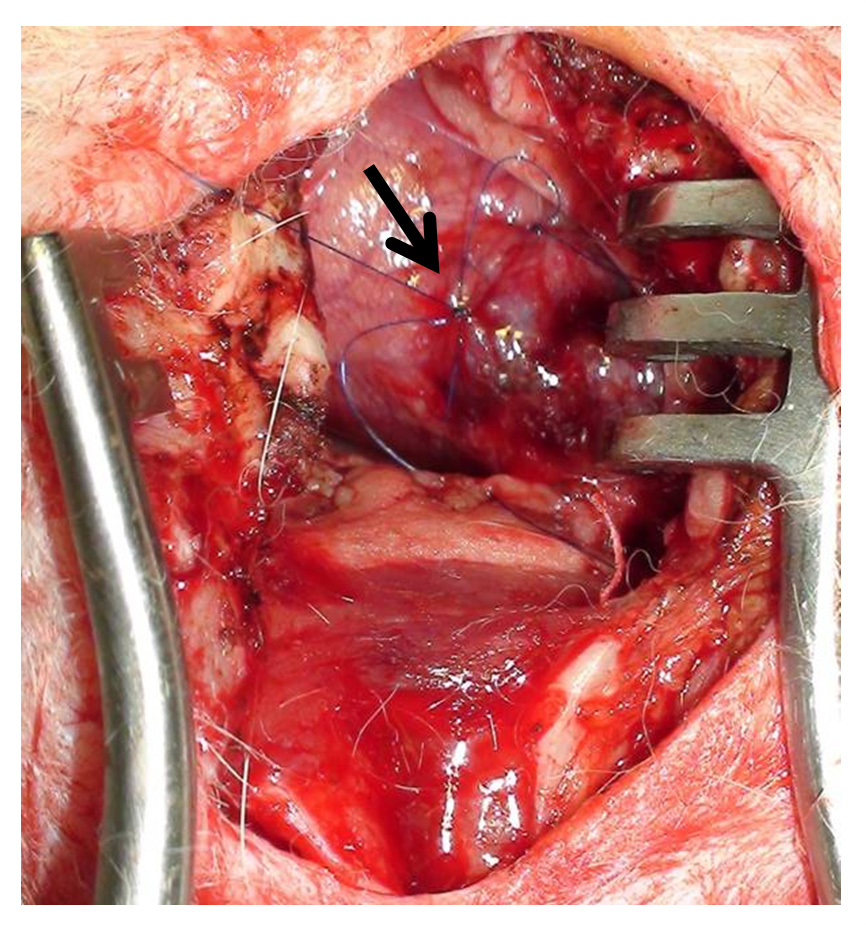
**Open chest surgical ligation EMI model**. Note the plain nylon suture knot (black arrow) placed around the distal LAD after heart exposure with a surgical mini median sternotomy incision.

A skin incision was made over the subxiphoid region and sternum after a povidone-iodine antiseptic scrub. The xiphoid process was carefully detached from the sternal part of diaphragm. A mini median stermotomy was performed carefully along the midline to avoid injury to the parietal pleura. The sternal edges were spread and an incision was made at the pericardiac sac to expose the left myocardial ventricular wall. An 18-gauge catheter was inserted into the left ventricle via the left ventricular apex for continuous measurement of hemodynamics. Immediately before coronary artery ligation, 1 mg/kg of lidocaine was administered intravenously to minimize potentially lethal ventricular arrhythmias. A 5-0 monofilament polyprolene suture was placed around the left anterior descending (LAD) coronary artery approximately 8-10 mm from its origin (Figure 1). The sternum, muscle layers and skin were then closed and rabbits were allowed to recover.

### Percutaneous closed-chest technique

**Figure 2:**
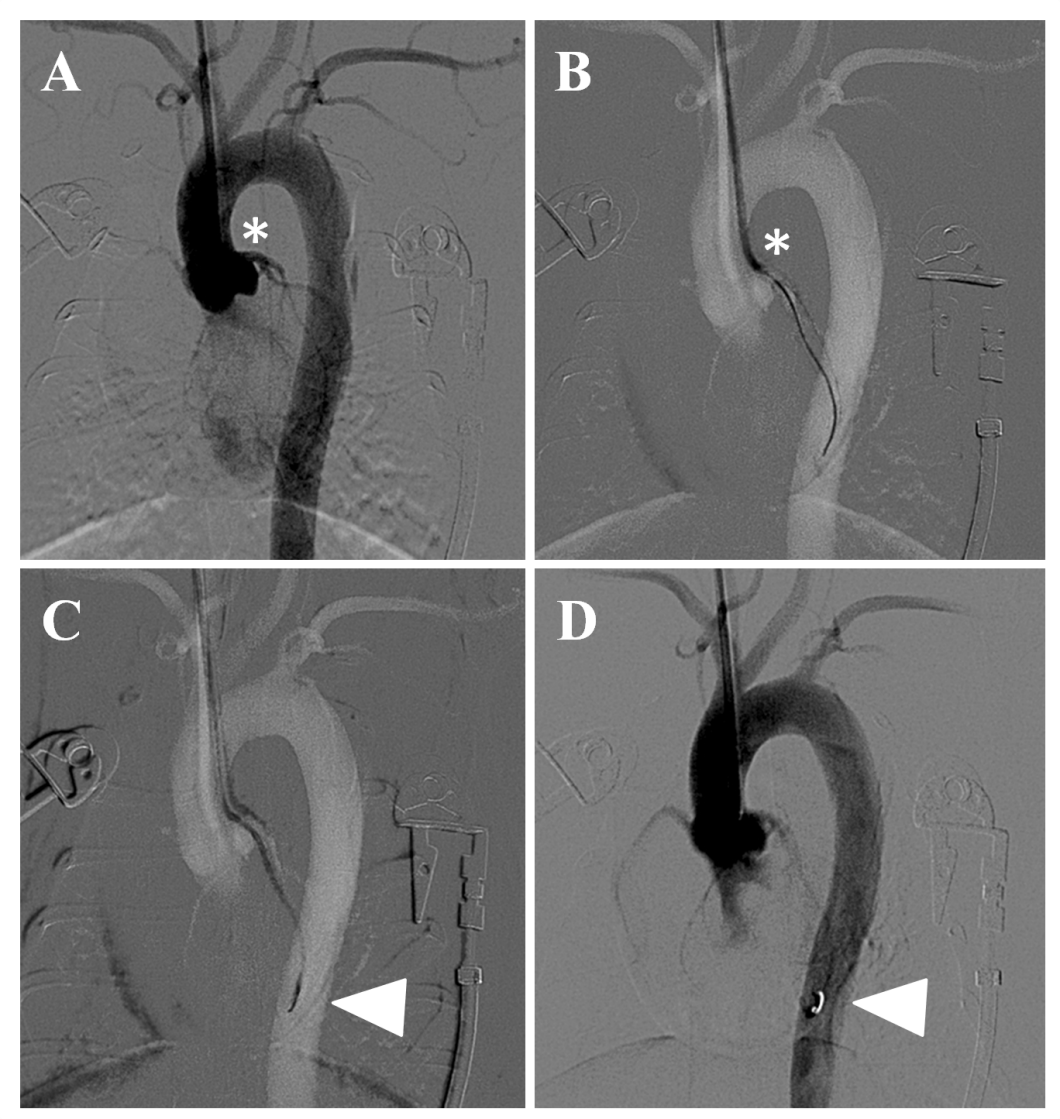
**Closed chest transauricualr embolization EMI model**. (A) An angled 4Fr hydrophilic cathater has been introduced retrograde to the aortic root after transauricular intra-arterial access of the right common carotid artery. Asterisk denotes the orifice of the LAD during contrast coronary angiogram obtained at 6 frames per second. (B) An 0.018 soft hydrophilic guidewire has been advanced in the distal LAD to guide insertion of a 2.4Fr Progreat microcatheter (Terumo, Japan). (C) The microcathater has been advanced to the level of the distal third segment of the LAD and beyond the origin of the diagonal branch and a 2 mm micro-coil is being delivered (white arrowhead). (D) Final contrast coronary angiogram shows micro-coil (white arrowhead) in situ after removal of the microcatheter (whole coronary catheterization and coil embolization procedure lasts < 3 min).

Cardiovascular monitoring was performed as previously described for the open procedure. To minimize coronary artery spasm and possible arrhythmias, a prophylactic dose of nitroglycerine (2 μg/kg i.v.) and lidocaine (1 mg/kg i.v.) was injected intravenously as recommended [9]. Antibiotic prophylaxis with cephalosporin was also administered (cefuroxime i.m.). Animals were immobilized in the supine position under c-arm fluoroscopy (Allura Xper FD20, Philips, Germany) and the left auricular dorsum was clipped, shaved and scrubbed with povidone-iodine. Transauricular intra-arterial access was gained as described previously [10]. Briefly, the central auricular artery was punctured with a standard 22-gauge intravenous catheter (Helmflon; Helm Pharmaceuticals, Hamburg, Germany). The central needle of the catheter was removed and diluted contrast agent (1:1) was infused to opacify the auricular artery and common and external carotid artery in a retrograde fashion. Then, under roadmap guidance, a 0.018-inch hydrophilic guide wire (V-18 control wire; Boston Scientific, Natick, MA) was carefully advanced into the carotid circulation and navigated down to the left ventricle. A 2- to 3-cm-long incision of the dermis was performed at the point of the initial puncture of the auricular artery and along the course of the guide wire in order to insert a 4-F, 0.018-inch guide wire-compatible vascular sheath (Bolton Medical, Villers-les-Nancy, France) after serial step-by-step dilations to remove the tight distal peripheral segment of the artery. After successful sheath insertion, each subject was heparinized with 100 IU heparin. A 4Fr angled hydrophilic catheter was introduced into the left ventricular cavity to measure the cardiac intraventricular pressure before and after EMI induction. A coronary angiogram was obtained at high magnification and 6fps acquisition rate to visualize the ostia and anatomy of the right and left coronary arteries (see Additional File 1 Video 1). Next, a 2.4Fr microcatheter (Progreat 2.4, Terumo, Japan) was advanced into the aortic root at the level of the Valsava sinus in order to engage the left coronary artery. The 4Fr angled catheter was positioned slightly higher so as to direct the microcatheter against the ostium of the left coronary artery. Superselective catheterization of the left anterior descending coronary artery was achieved with the help of a soft-tipped angled 0.018 hydrophilic wire (the guidewire supplied with Progreat 2.7Fr, Terumo, Japan) that was advanced almost at the level of the cardiac apex. Finally, the 2.4Fr Progreat microcatheter was carefully slided over its guidewire and introduced approximately at the mid-level of the left coronary artery so that its tip was positioned beyond the orifice of the diagonal branch but not too deep to avoid mechanical obstruction. EMI was produced with prompt delivery of a 2 × 10 mm micro-coil (2 × 25 micro-coil, Balt Extrusion, France; coil was manually cut in almost half) (Figure 2). All sequential steps of coronary microcatheter cannulation, delivery of the micro-coil, and removal of the microcatheter were performed in a gentle and extra cautious manner to coronary spasm and/or dissection (see Additional File 2 Video 2). After EMI induction, the sheath was removed and hemostasis was achieved with clothespin compression for 5-10 minutes.

### Blood biochemistry and histopathology

Blood samples were taken and levels of myocardial enzymes were measured in blood serum. Aspirate aminotransferase (AST), lactate dehydrogenase (LDH), creatine kinase (CK), the MB isoenzyme of creatine kinase (CK-MB) and troponin were measured with microparticle enzyme immunoassay (ΜΕΙΑ- AxSYM Abbott) and Liaison assay before and at 6, 12 and 24 hours after EMI induction to confirm myocardial infarction. Four weeks after endovascular or surgical EMI induction, the rabbits were anesthetized again as described previously. A median sternotomy was performed and adhering tissue was carefully removed around the myocardium. The rabbits were anticoagulated with 1,000 IU heparin intravenously to prevent clotting. Hearts were then dissected and excised with the aortic root. Following macroscopic examination, 3 mm-thick sections were taken at the greatest dimension of visible "gray-white" post-infarct fibrotic areas and then fixed in 10% formalin and embedded in paraffin. Four-μm sections from paraffin-embedded blocks were stained with hematoxylin-eosin (H&E) and Masson trichrome staining for further histopathological analysis.

### Statistical analysis

Discrete variables were expressed as counts and percentages and continuous variables were given as means ± standard deviation. The unpaired t test was used to test the significance of difference of variables. The Welch's correction was applied in case of comparisons with unequal sample variances. Statistical analysis was performed with use of the GraphPad Prism statistical software package (version 5; GraphPad Software, La Jolla, California, USA). The threshold of statistical significance was set at a p value of 0.05.

## Results

A total of 38 rabbits were used in the surgical open-chest group A (n = 17) and percutaneous closed-chest group B (n = 21). There were 5 deaths in the surgical procedure group equivalent to an overall mortality of 29.4% (5/17). Two of the subjects died immediately after the operation, one due to ventricular fibrillation and one because of ligation of LAD too proximally to its origin. The other three died 2-10 days after surgical procedure, two of unknown causes and one because of sepsis.

On the other hand, 9 deaths were recorded in the percutaneous closed-chest group B. The most common cause of death was ventricular fibrillation, electromechanical dissociation and progressive cardiogenic shock, all of which were usually associated with very proximal coil placement covering the ostium of the diagonal branch that provides critical perfusion of the atrioventricular node. One rabbit died because of coronary artery dissection. Another one died due to severe artery spasm because of long-lasting residence of the microcatheter inside the left coronary artery. Two rabbits suffered a late death during the first 10 days after EMI induction. The overall mortality rate was 42.9% (9/21). There was no difference in the mean body weight between the two groups before LAD occlusion and at the day of euthanasia 4 weeks later.

### ECG and biochemistry results

ECG of all rabbits was normal at baseline. ST segment elevation, an indicator of severe ischemia or an ongoing myocardial infarction, was confirmed in each animal following occlusion of the LAD without any statistically significant differences between the 2 treatment groups (Table 1). Of note, there was immediate sharp ST-segment elevation aftercoronary ligation in group A, while ST elevation was delayed and recorded approximately 15-20 min after percutaneous coronary embolization in group B. Four weeks after myocardial infarction ECG showed abnormal Q waves in all surviving animals of both groups. Myocardial ischemia was also confirmed with a significant increase of AST, LDH, CPK, CPK-MB and troponin after occlusion of the coronary artery. There were no statistically significant differences in myocardial-enzyme changes 24 hours after LAD occlusion between the two groups.

**Table 1:** Experimental results

Table 1.	Closed-chest transauricular coronary embolization	Open-chest surgical coronary suture ligation	
**NZW rabbits**	n = 21	n = 17	*P *

Weight (kg) at baseline	3.15 ± 0.25	3.13 ± 0.23	0.80
Weight (kg) at 4 weeks	3.30 ± 0.3	3.27 ± 0.28	0.75
Mortality (%)	9/21 (42.9%)	5/17 (29.4%)	0.20

**ECG outcomes**			*P*

Abnormal ECG	15-20 min	Immediately	n/a
ST elevation (mm)	2.01 ± 0.84	1.90 ± 0.71	0.68

**Blood biochemistry**			*P*

Troponin (ng/ml) baseline	0.06 ± 0.06	0.05 ± 0.09	0.70
Troponin (ng/ml) (24 h)	47.6 ± 64.6	44.5 ± 57.7	0.88
AST baseline(U/L)	36.8 ± 21.4	33.2 ± 20.4	0.60
AST (U/L)*	110.0 ± 87.3	105.8 ± 87.6	0.88
LDH baseline(U/L)	169.4 ± 112.2	176.2 ± 101.8	0.85
LDH(U/L)*	967.4 ± 976.9	913.3 ± 820.8	0.85
CPK baseline (U/L)	1,072.0 ± 367.7	1,007.8 ± 287.2	0.55
CPK (U/L)*	6,725.8 ± 4,642.1	6,386.3 ± 3,921.4	0.81
CK -(MB) baseline (ng/ml)	0.87 ± 0.42	0.82 ± 0.33	0.68
CK -(MB) (ng/ml)*	2.88 ± 1.68	2.78 ± 1.37	0.84

**Post-infarct outcomes**			*P*

Abnormal Q waves	All surviving animals	All surviving animals	n/a
Infarct size, cm (range)	0.92 ± 0.54 (0.3-2.0)	0.86 ± 0.35(0.5-1.7)	0.68
Baseline LVEDP (mmHg)	6.7 ± 1.8	6.2 ± 1.7	0.39
4-week LVEDP (mmHg)	12.9 ± 2.3	12.7 ± 2.0	0.78

Continuous variables are presented as mean ± SD.

*Maximum recorded values of serum enzymes are presented.

### Cardiac function and hemodynamic outcomes

Cardiac function was assessed by recording left ventricular end-diastolic pressure (LVEDP) that offers an indication of heart systolic function. LVEDP measurements were taken from an intraventricular catheter before, immediately after EMI and also before animal euthanasia. EMI caused a significant increase in LVEDP in all infarcted groups as evidence of myocardial dysfunction. LVEDP increased to a similar extent in both groups measuring 12.7 ± 2.0 vs 12.9 ± 2.3 mmHg at 4 weeks before euthanasia, compared to 6.2 ± 1.7 vs 6.7 ± 1.8 mm Hg at baseline for the surgical group A and the percutaneous group B, respectively.

### Post-infarct histology

**Figure 3:**
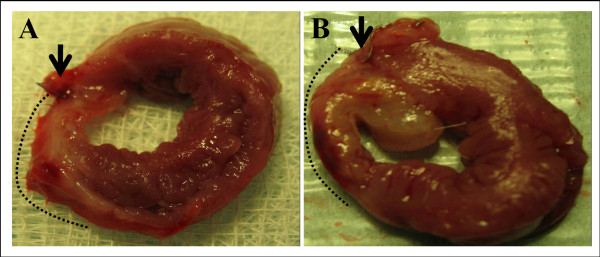
**Gross histology of harvested hearts**. Transverse 3 mm sections were taken at the greatest dimension of visible post-infarct fibrotic areas. Dotted black line denotes the "gray-white" myocardial infarcted zone in each specimen. Note (A) the plain suture (black arrow) in a subject treated with open-chest ligation and (B) the platinum micro-coil (black arrow) in a subject treated with transauricular transcatheter LAD embolization.

**Figure 4:**
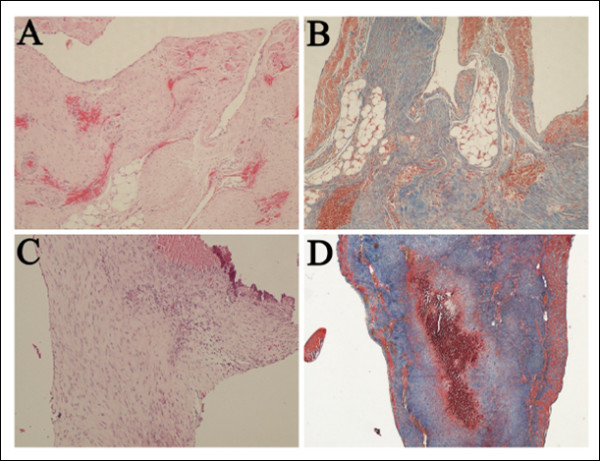
**Histological examination**. (A, C) Hematoxylin&Eosin and (B, D) Masson trichrome stain of myocardial infarction in representative cases of rabbits treated with the open-chest surgical method (A, B) and the percutaneous closed-chest technique (C, D).

Histolopathological analysis of heart sections confirmed the presence of myocardial infarction in both groups. Granulation tissue showing new blood vessels, infiltration by lymphocytes, plasma cells and histiocytes, as well as collagen deposition was identified at the site of myocardial infarction. Infarct size was measured on sections stained with H&E and Masson trichrome that were taken at the greatest dimension of macroscopic gray-white areas. Infarct size measured 0.86 ± 0.35 cm (range, 0.5-1.7 cm) in the group treated with the conventional surgical method and 0.92 ± 0.54 cm (range, 3-2 cm) in the rabbits of the minimally invasive method (Figures 3 and 4). There was no statistically significant difference in the size of the infarct between the two groups. All outcome variables are outlined in detail in table 1.

## Discussion

In this study, we have developed a solely percutaneous minimally invasive method to occlude the left anterior descending artery and produce experimental myocardial infarction by employing interventional catheterization techniques and fluoroscopic guidance. Transauricular percutaneous EMI may avoid thoracotomy, pericardial incision and other local and systemic side effects of major surgery and may therefore achieve a more physiological model of EMI. From a historical perspective, except from LAD ligation with plain sutures [3,4,11], which has been the standard surgical technique of EMI ever since 1881 [12], there are also open-chest techniques where coronary blood flow restriction may be established by circumferential placement of an ameroid constrictor [13], a non-elastic Dacron stripe [14] or compression by an arterial puncture needle [15]. Moreover, induction of coronary artery occlusion by cryoinfarction [16] or electrolytic coronary injury and thrombosis [17] has been reported. Researchers have also established closed-chest techniques of EMI with the application of various different embolic materials like plugs [18], balloons [19], coils [20], beads [21], microspheres [22], or even gelatine sponges [23]. Myocardial necrosis has been also achieved by intracoronary injection of irrigative chemical substances like ethanol or ethyl-alcohol [24] and thrombosis of the LAD artery [25].

Most importantly, closed-chest EMI may induce more physiologically relevant myocardial ischemia for the study of cardiac ischemic recovery and remodeling, as well as for pharmacological investigations of novel cytokine-, gene- or cell-based proangiogenic therapies (heart therapeutic angiogenesis) [3]. There is immediate angiographic documentation of LAD stenosis or occlusion and because of its endovascular nature the options of studying myocardial reperfusion, preconditioning and thrombolysis are inherently offered. On the other hand, depending on the technique, the exact location and length of coronary artery occlusion, the overall volume of myocardial necrosis, the rate and time of spontaneous thrombolysis, the extent of endothelial damage and the amount of reflux of the injected agent into proximal coronary arteries and side branches cannot be easily controlled nor estimated [24,26]. In order to achieve a fairly reproducible model of EMI we used a very short micro-coil delivered to the distal half of the rabbit LAD and beyond the origin of the diagonal branch. Obstruction of the latter would lead to fatal cardiac arrythmias. In addition, the proposed method requires the mastering of advanced technical skills in microcatheter manipulation and transcatheter embolization, employs fluoroscopic guidance and use of expensive instruments is necessary.

To date, myocardial ischemia has been widely studied in different species of animals. Big animals such as the swine, dog, sheep and smaller like the rabbit and the rat have been extensively used in EMI research [3,27]. Large animals may be more suitable for cardiovascular studies because of their anatomy and physiology, but are often cost-prohibitive and have the disadvantage of costly housing and difficulties in handling and manipulation. The rabbit was our choice because of its small size, universal availability and low cost. It has been also shown that the rabbit heart bears similar anatomy and pathophysiology to the human heart in that both recruit poor collaterals after ischemia [27,28], thus making them well suited for EMI studies.

Derugin et al. described a method of occluding the left circumflex coronary artery with a small, nonmagnetic coil that was fluoroscopically guided via the left femoral artery in seven rabbits [29]. However, in this study there was no control group available. Edwards and his colleagues also reported a closed-chest technique of myocardial infarction in the rabbits by placement of a 0.36 mm thrombogenic coil in the circumflex artery via the right common carotid artery that was accessed through median cervical incision [20]. The herein proposed methodology is solely percutaneous and was also compared with the gold standard of experimental myocardial infarction induced by LAD ligation with a plain suture. A micro-coil was advanced in the coronary artery via a transauricular endovascular access, which was accomplished by percutaneous catheterization of the central auricular artery of the rabbit. From a technical perspective, LAD micro-coil embolization has the primary advantage of controlled and precise artery occlusion under high-quality fluoroscopic guidance. In the present EMI model, insertion of a very small micro-coil is proposed in order to be accommodated by the very small caliber distal coronary artery that has a diameter of less than 1 mm.

Traditionally, intra-arterial access in an animal is achieved by surgical cutdown of the femoral or cervical arteries and veins, which may be finally ligated and thrombosed, limiting future reuse of the vessel. The transauricular approach is a safe, quick, minimally invasive, and highly successful technique to achieve central endovascular access in the rabbit experimental model. It obviates surgical cutdown and sacrifice of the femoral and cervical vessels. Moreover, the subjects experience less pain, bleeding complications, and wound infections [10,30,31]. A relative limitation of the transauricular approach is the destruction of the peripheral segment of the auricular artery during sheath insertion. However, to our experience this is always followed by uneventful healing with development of granulation tissue at the dorsum of the auricle. Alternatively, the rabbit transauricular intra-arterial access may be directly gained with a microcatheter in a sheathless way as proposed elsewhere [31].

In comparison with the surgical model of EMI with plain suture LAD ligation, the percutaneous closed-chest model demonstrated almost equivalent increase of troponin and other cardiac enzymes in blood serum after myocardial infarction without any statically significant differences between the two groups. Regarding ECG changes, the ST-segment elevated immediately after surgical ligation of left anterior descending artery, whereas in the percutaneous technique the ST elevation was recorded 15-20 min after coil embolization of the coronary artery, probably because of gradual coil thrombosis and delayed total thrombotic occlusion of the artery. ECG showed abnormal Q waves in all surviving animals of both groups. Moreover, cardiac function as estimated by LVEDP was similarly disturbed in both groups 4 weeks after EMI induction. Finally, there was no statistically significant difference in the myocardial infarcted area between the 2 groups. The overall mortality was higher in the minimally invasive model in comparison with the surgical model, probably due to the steep learning curve of the embolization technique and the very small caliber of the target vessel that could abruptly spasm or thrombose. However, with amassed experience and careful choice of size and shape of catheters and drugs periprocedural mortality may be reasonably reduced. We would anticipate more optimal results with the use of finer microcatheters and guidewires like the ones suitable for neurovascular interventions. In conclusion, the herein presented minimally invasive model of percutaneous transauricular coronary coil embolization technique may be a reliable and reproducible platform for the experimental study of myocardial ischemia.

## Conflicts of interest

The authors declare that they have no competing interests.

## Authors' contributions

KK and SM conceived the study, performed the experimental protocols, collected the data, performed the data analysis and drafted the manuscript. EK participated in the surgical experimental protocol and in data collection. VB performed the histopathological analysis of the specimens. FK performed the biochemical analysis of the specimens. DK and AD participated in the transauricular experimental protocol and performed the statistical analysis. DD and DS were responsible for the overall design of the experimental protocols and coordinated the study. All authors have read and approved the final manuscript.

## Supplementary Material

Additional file 1 **Video 1. Baseline coronary angiogram**. Baseline transcatheter contrast coronary angiogram in the New Zealand White rabbit after percutaneous transauricular intra-arterial access (6fps acquisition rate). Click here for file

Additional file 2 **Video 2. Post-embolization coronary angiogram**. Transcatheter contrast coronary angiogram in the New Zealand White rabbit after distal micro-coil embolization of the left anterior descending coronary artery (6fps acquisition rate). Click here for file
